# Pathways of host cell exit by intracellular pathogens

**DOI:** 10.15698/mic2018.12.659

**Published:** 2018-10-18

**Authors:** Antje Flieger, Freddy Frischknecht, Georg Häcker, Mathias W. Hornef, Gabriele Pradel

**Affiliations:** 1 Division of Enteropathogenic Bacteria and Legionella, Robert Koch Institute, Wernigerode, Germany.; 2 Center for Infectious Diseases, University of Heidelberg, Germany.; 3 Institute of Medical Microbiology and Hygiene, Medical Center - University of Freiburg, Germany.; 4 Institute of Medical Microbiology, RWTH Aachen University Hospital, Germany.; 5 Division of Cellular and Applied Infection Biology, Institute of Biology II, RWTH Aachen University, Germany.

**Keywords:** host cell, egress, exit, pathogen, vacuolar escape, compartment, programmed cell death, membrane lysis.

## Abstract

Host cell exit is a critical step in the life-cycle of intracellular pathogens, intimately linked to barrier penetration, tissue dissemination, inflammation, and pathogen transmission. Like cell invasion and intracellular survival, host cell exit represents a well-regulated program that has evolved during host-pathogen co-evolution and that relies on the dynamic and intricate interplay between multiple host and microbial factors. Three distinct pathways of host cell exit have been identified that are employed by three different taxa of intracellular pathogens, bacteria, fungi and protozoa, namely (i) the initiation of programmed cell death, (ii) the active breaching of host cellderived membranes, and (iii) the induced membrane-dependent exit without host cell lysis. Strikingly, an increasing number of studies show that the majority of intracellular pathogens utilize more than one of these strategies, dependent on life-cycle stage, environmental factors and/or host cell type. This review summarizes the diverse exit strategies of intracellular-living bacterial, fungal and protozoan pathogens and discusses the convergently evolved commonalities as well as system-specific variations thereof. Key microbial molecules involved in host cell exit are highlighted and discussed as potential targets for future interventional approaches.

## INTRODUCTION

Infectious diseases caused by viruses, bacteria, fungi, or parasites still represent a major cause of morbidity worldwide and account for almost 50,000 deaths every day [1]. During the course of infection, many bacterial, fungal and protozoan pathogens rely on a life-cycle phase, during which they parasitize host cells, typically but not always contained by membranous vacuoles. The intracellular lifestyle provides several advantages, including accessibility to nutrients and evasion from the human humoral and cellular immune system. Research during the last decades has revealed manifold examples illustrating the intricate and sophisticated strategies of microorganisms to invade their host cells and to manipulate the intracellular environment in order to promote microbial survival and to evade destruction by the host immune system (reviewed in [2]). Although crucial to ensure life-cycle progression, and, in consequence, dissemination and transmission, host cell exit has hitherto remained largely unresolved. Recent results, which were mainly gained from studies on *Listeria, Toxoplasma* and *Plasmodium*, provide firm evidence that host cell exit, initially viewed as a largely passive process, represents an active and dynamic interplay between the pathogen and the infected cell. Three major exit pathways, (i) the induction of programmed cell death (PCD), (ii) the active breaching of host cell-derived membranes, and (iii) the induced membrane-dependent exit without host cell lysis, have been identified. The pathways follow a spatially and temporally defined coordinated process and involve the interaction of pathogen- and host-derived effector molecules (reviewed in [3-7]).

Host cell exit by human pathogens is typically linked to tissue inflammation, organ dysfunction, and host-to-host transmission and thus significantly contributes to disease burden and the epidemiology of infectious diseases. Hence, a detailed knowledge of the underlying mechanisms of microbial cell exit is essential to understand the pathogenesis of infectious diseases. This is demonstrated by the critical role of exit-associated microbial effector molecules for the survival and spread of pathogens. An example is the recent finding that chemical inhibition of plasmepsin (PM) proteases can interrupt the egress of malaria parasites from the enveloping erythrocyte [8, 9]. The present review therefore aims at attracting the well-deserved attention to this essential and understudied life-cycle phase of intracellular pathogens. We will explore the diverse exit strategies of intracellular-living bacterial, fungal, and protozoan pathogens, and discuss key microbial and host effector molecules. Further, we will assess the cellular and molecular aspects of microbial host cell exit and evaluate potential targets for future interventional strategies to combat infections by intracellular pathogens.

## EXIT PATHWAYS

**Figure 1:**
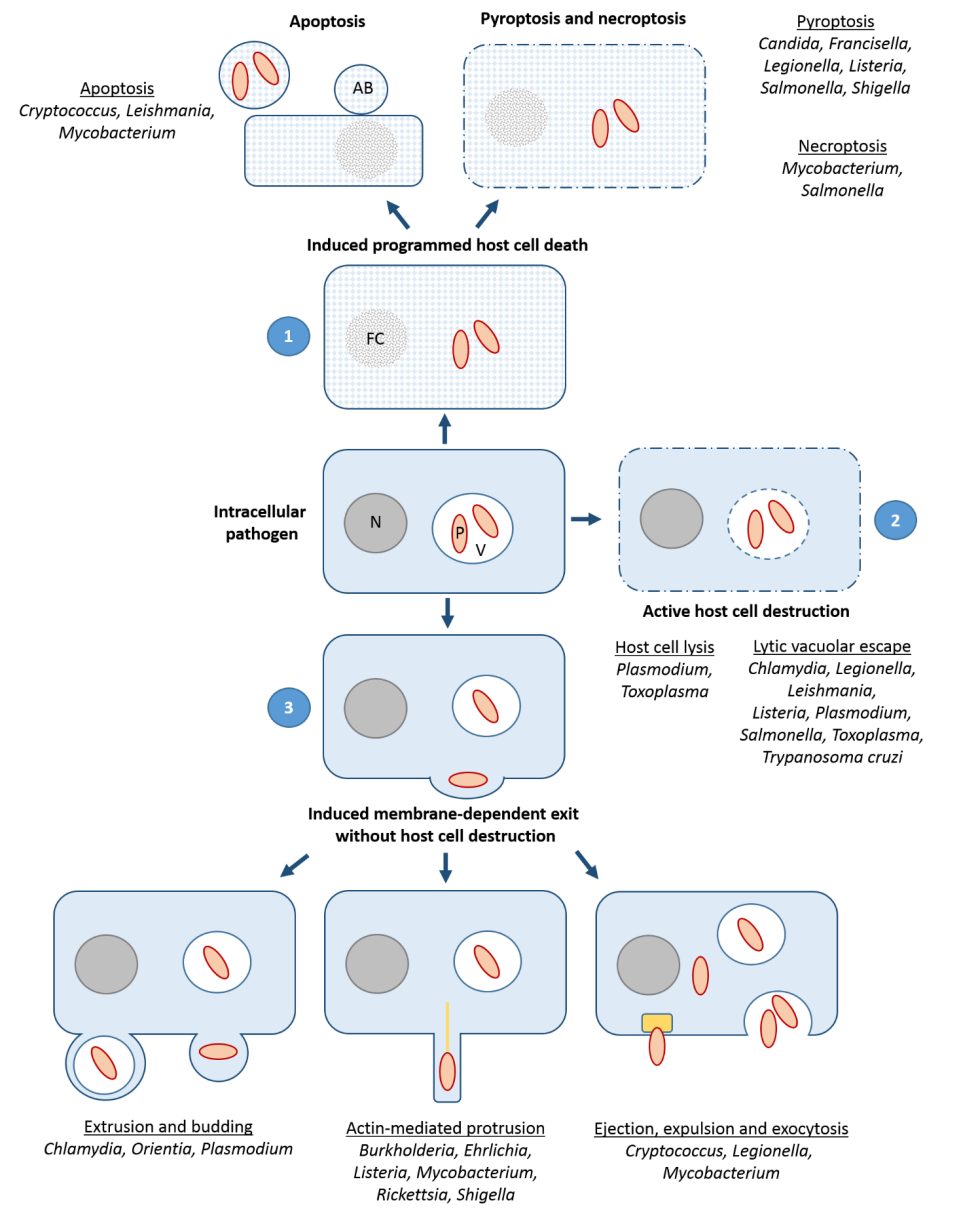
FIGURE 1: The three strategies of host cell exit. **(1)** Induction of programmed cell death, including the non-lytic apoptosis and the lytic necroptosis and pyroptosis pathways; **(2)** Active host cell destruction, comprising breaching of host cell membranes such as the vacuolar and/or the host cell plasma membrane; **(3)** Induced membrane-dependent exit without host cell destruction, e.g. by actin tail (yellow line)- mediated protrusions, extrusions, budding, exocytosis, expulsion or ejection via an ejectosome (yellow box). Pathogens for which distinct pathways were demonstrated are indicated. AB, apoptotic body; FC, fragmented chromatin; N, nucleus; P, pathogen; V, vacuole.

Current data indicate that at least three principal exit pathways are used by intracellular pathogens (outlined in Fig. 1) (reviewed in [3-7]): (i) PCD including the non-lytic apoptosis and the lytic necroptosis and pyroptosis pathways, employed by bacterial, fungal and protozoan pathogens; (ii) the active breaching of host cell-derived membranes such as the endosomal, the vacuolar and/or the host cell plasma membrane, as shown for a variety of bacterial and protozoan parasites; (iii) the induced membrane-dependent exit without host cell lysis, e.g. via actin-based protrusions, extrusions, budding, exocytosis, expulsion or ejection, as has been demonstrated for some bacteria, for the yeast *Cryptococcus* and for *Plasmodium*. While the first two pathways ultimately result in killing of the host cell, the third pathway in general leaves the host cell intact. Intriguingly, recent work suggests that the majority of intracellular pathogens is able to utilize more than one of these pathways, dependent on life-cycle stage, environmental factors and/or host cell type.

### Induction of programmed host cell death

PCD represents an intrinsic, regulated process of cell death with typical morphological changes and an important contribution to tissue ontogeny, cellular immunity and organ homeostasis. Different forms of PCD, i.e. apoptosis, necroptosis and pyroptosis, have been described that are initiated by specific signals and signal transduction cascades and that exhibit defined phenotypic characteristics (reviewed in [10]). PCD is also observed during infection, where it allows exfoliation and removal of infected cells. Here, a typical distinction is the one between lytic and non-lytic cell death, which has a profound impact of the immune response and, in consequence, on the course of infection. Lytic death is associated with early cell membrane permeabilization and release of cell debris, whereas non-lytic cell death maintains the surface integrity of the dying cell until its uptake by a phagocyte. Non-lytic cell death is largely confined to apoptosis whereas both pyroptosis and necroptosis cause early loss of plasma membrane integrity. Notably, even late stage apoptosis has a programmed lytic component, referred to as secondary necrosis. Accumulating evidence suggests that microbial targeting of PCD-inducing signal transduction pathways represents a cell exit strategy for a number of pathogens (Fig. 1).

#### Apoptosis

Apoptosis represents an evolutionarily conserved mechanism that contributes significantly to organ development and tissue homeostasis. Apoptosis also plays a prominent role in preventing pathogen progeny, as previously observed during viral infections [11]. It is characterized by typical morphological changes, including the disintegration of the apoptotic cell into condensed apoptotic bodies, which are subsequently taken up by phagocytes, particularly macrophages. Via uptake of these apoptotic bodies, viable pathogens could be transferred to a phagocytic cell in a non-inflammatory context. Apoptosis would therefore not release the intracellular bacterium to the extracellular space, but would rather lead to its re-uptake and thus cell-to-cell spread. The pathogen will remain within a membrane-enclosed particle and end up in a phagosome, where it has to deal with multiple degradative mechanisms. This is illustrated by the phagocyte-infecting gram-positive bacterium *Mycobacterium tuberculosis*, which is taken up within apoptotic bodies via efferocytosis [12]. *M. tuberculosis* is subsequently killed by the macrophage. In contrast, the protozoan parasite *Leishmania spp*., the causative agent of the vector-borne tropical disease leishmaniosis, is internalized by macrophages packed in apoptotic bodies and subsequently establishes itself in the phagosomal compartment [13, 14]. Furthermore, the fungal pathogen *Cryptococcus neoformans*, which can cause infections of a number of organs, including the lung and central nervous system, can exit the cell through lytic and non-lytic mechanisms, and the lytic mechanism has been found to be of apoptotic nature, even though lysis does not normally occur during apoptosis [15].

#### Necroptosis

Necroptosis shows phenotypic characteristics of necrotic cell death (thus also termed programmed necrosis) and is induced by a specific signaling pathway that includes the receptor-interacting protein kinase 3 (RIP3/RIPK3) and the mixed lineage kinase domain like pseudokinase (MLKL) (reviewed in [16, 17]). It has to be kept in mind that most mechanistic studies of necroptosis use agonists of deathreceptor signalling to induce necroptosis. In this situation, necroptosis only occurs if caspase-8, which belongs to a group of PCD-related cysteine-aspartic proteases, is inhibited via chemical or gene-knock out approaches. Although such approaches have improved our understanding, this type of experimental design is not physiological. The role of necroptosis during infection therefore requires further investigations.

Necroptosis has been observed during infection with the facultative intracellular gram-negative bacterium *Salmonella enterica* serovar Typhimurium (*S*. Typhimurium), a pathogen causing gastroenteritis in humans following ingestion of contaminated food or water. Following penetration of the intestinal mucosal barrier, *S*. Typhimurium is taken up by macrophages and induces necroptosis [18]. Unexpectedly, this induction depends on type I interferon and the inhibition of necroptosis reduces the bacterial organ load [19]. However, it remains unresolved whether this is because cell exit is blocked or because macrophages survive and kill the bacterium.

A number of studies have investigated the role of necroptosis during mycobacterial infection. The most intriguing finding is that death of neutrophils that have engulfed virulent *M. tuberculosis* is instrumental to the transfer of the bacteria into macrophages, where they proliferate and induce persistent infection [20]. Cell death in neutrophils is induced via reactive oxygen species (ROS). Because the study was performed in human cells where the possibilities of genetic modification are limited, it is not clear though, if death of the neutrophil mechanistically represents necroptosis. As shown, chemical inhibition of neutrophilic ROS production prevents cell death. In addition, inhibition of RIPK1 (which is often involved in necroptosis) reduced bacterial transfer [20].

Another possible way of inducing necroptosis is through the cytosolic protein Z-DNA-binding protein ZBP1 (also known as DAI). Upon binding of specific conformations of nucleic acids, ZBP1 can activate the RIPK3/MLKL signalling axis [21]. While this has only been described for viruses so far [22], it would not be surprising if ZBP1 also had a function in bacterium- or parasite-induced PCD, given the overlap of viral and bacterial pattern recognition. In fact, ZBP1 was upregulated in *M. tuberculosis*-infected macrophages [23], although no specific function has been assigned.

#### Pyroptosis

Pyroptosis involves pores in the plasma membrane formed by members of the gasdermin family, following their proteolytic cleavage by caspases. Pyroptosis thus requires the proteolytic activity of at least one caspase and a number of caspases have been found to be able to induce gasdermin-cleavage and pyroptosis, e.g. caspase-1 and -11 (caspase-11 is only found in mice, its orthologues in humans are caspase-4 and -5), but also to some degree by the apoptotic caspase-3 [10, 24-26]. These caspases are activated by multiprotein complexes known as inflammasomes that are formed by oligomerization of caspase-adapter proteins (reviewed in [27, 28]).

Intriguingly, pyroptotic caspases can be activated upon recognition of microbial ‘patterns’ in the cytosol. An example is the recognition of cytosolic lipopolysaccharide by caspase-11 (reviewed in [28]). A substantial number of bacteria have been found to activate pyroptosis, among them *Legionella, Francisella, Shigella, Salmonella* and *Listeria* (reviewed in [3, 4, 7]). Previous investigations of pyroptosis during microbial infection have focused on its potential role in host defense rather than microbial host cell exit. The deletion of various components of pyroptosis signaling enhances the sensitivity against bacteria; the deletion of caspase-11 for instance sensitizes host cells and mice against enteric *Salmonella* infection [29] as well as against infection with *Legionella pneumophila* [30].

Another case in point is the opportunistic yeast pathogen *Candida albicans*, which induces pyroptosis in macrophages instrumental to facilitate release of the microbe [31]. It therefore seems very likely that pyroptosis, while involved in the antimicrobial host response and the initiation of inflammation, in addition plays a substantial role in cell exit. Pyroptosis is associated with secretion of proinflammatory cytokines that drive inflammation and pyroptotic PCD is expected to further augment inflammation through the release of both microbial components, making them available to the immune system and constituents of the dying cell (damage-associated molecular patterns; reviewed in [32]). Although inflammation is primarily a defense reaction with detrimental consequences to the pathogen, its downstream effects such as changes in the metabolism or influx of immune cells might actually favor growth and tissue spread of the pathogen. Microbedirected skewing of the immune response by specific signals might further diminish the antimicrobial effect and enhance the pathogen’s benefit. Notably, pyroptosis, unlike apoptosis and probably necroptosis, is limited to specific cell types, such as macrophages, since not all cells express or can activate inflammasome components.

### Active host cell destruction

Active host cell destruction describes an exit process, during which microbial molecules penetrate or perforate membranes, such as the host cell plasma membrane or the membrane of the vacuolar compartment, in consequence destroying host cell and compartment, respectively (Fig. 1). Exceptionally, active destruction can include the walls of intra- or extracellular pathogen-containing cysts. Particularly, three types of proteins were described that mediate active host cell lysis, i.e. proteases, phospholipases, and pore-forming proteins (Table 1).

**Table 1:** Microbial key factors of host cell exit.

**Type**	**Factor**	**Pathogen (stage)**	**Pathway**	**Function**
**Protease**	CPAF	*Chlamydia trachomatis*	Lytic vacuolar escape	Processes host cell proteins, e.g. vimentin and LAP-1
	SUB1	*Plasmodium falciparum* (RBC merozoite)	Host cell lysis	Processes effectors, e.g. SERA5, SERA6, MSP1
	SERA5	*Plasmodium falciparum* (RBC merozoite)	Host cell lysis	Protease-like w/o activity, unknown regulatory function
	SERA6	*Plasmodium falciparum* (RBC merozoite)	Host cell lysis	Spectrin cleavage, suggested to mediate destabilization of RBC cytoskeleton
	SERA5	*Plasmodium berghei* (oocyst)	Cyst destruction	Involved in sporozoite egress from the oocyst, unknown function
	PM VIII	*Plasmodium berghei* (oocyst)	Cyst destruction	Involved in sporozoite egress from the oocyst, unknown function
	PMX	*Plasmodium falciparum* (RBC merozoite)	Host cell lysis	Processing of effectors, e.g. SUB1
**Phospholipase/Cholesterol Acyltransferase**	PI-PLC PlcA	*Listeria monocytogenes*	Lytic vacuolar escape	Suggested to stalling autophagosome formation during vacuolar escape
	PC-PLC PlcB	*Listeria monocytogenes*	Lytic vacuolar escape	Suggested to stalling autophagosome formation during vacuolar escape
	PlaA	*Legionella pneumophila*	Lytic vacuolar escape	Involved in vacuolar membrane rupture, counteracted by SdhA
	SseJ	*Salmonella enterica serovar* Typhimurium	Lytic vacuolar escape	Involved in SCV membrane rupture, counteracted by SifA
	PbPL	*Plasmodium berghei* (liver stage merozoite)	Lytic vacuolar escape	Involved in PVM rupture
	LCAT	*Toxoplasma gondii* (tachyzoite)	Host cell lysis	Involved in PVM rupture, unknown function
**Poreformer/Cytolysin**	LLO	*Listeria monocytogenes*	Lytic vacuolar escape	Lyses membranes of primary and secondary vacuoles
	ESAT-6	*Mycobacterium tuberculosis*	Lytic vacuolar escape	Lyses vacuolar membrane
	CFP-10	*Mycobacterium tuberculosis*	Lytic vacuolar escape	Lyses vacuolar membrane
	T3SS1	*Salmonella enterica* serovar Typhimurium	Lytic vacuolar escape	Involved in vacuolar escape, unknown function
	IpaB	*Shigella flexneri*	Lytic vacuolar escape	Formation of pore complex in vacuolar membrane, cholesterol binding
	IpaC	*Shigella flexneri*	Lytic vacuolar escape	Formation of pore complex in vacuolar membrane, cholesterol binding
	IpaD	*Shigella flexneri*	Lytic vacuolar escape	Suggested to regulator formation of pore complex in vacuolar membrane
	Leishporin	*Leishmania*	Lytic vacuolar escape	Involved in phagolysosomale escape, unknown function
	PPLP1	*Plasmodium falciparum* (sporozoite)	Lytic vacuolar escape	Perforation of transient vacuolar membrane
	PPLP2	*Plasmodium falciparum/berghei* (gametocyte)	Host cell lysis	Perforation of the RBCM
	Tc-Tox	*Trypanosoma cruzi* (metacyclic trypomastigote)	Lytic vacuolar escape	Involved in vacuolar escape, unknown function
**Non-lytic egress**	Sec14, Plb1	*Cryptococcus neoformans*	Vomocytosis	Non-lytic escape from macrophages and amoebae
	LepA	*Legionella pneumophila*	Endocytosis	Non-lytic escape from amoebae
	LepB	*Legionella pneumophila*	Endocytosis	Non-lytic escape from amoebae
	ESAT-6	*Mycobacterium tuberculosis*	Ejection	Non-lytic escape from amoebae
	ESX-1	*Mycobacterium tuberculosis*	Ejection	Non-lytic escape from amoebae
**Cytoskeleton modulator**	BimA	*Burkholderia*	Protrusion	Involved in actin tail formation, mimicry of Ena/VASP actin polymerases
	ActA	*Listeria monocytogenes*	Protrusion	Involved in actin tail formation, WASP mimicry
	InlC	*Listeria monocytogenes*	Protrusion	Involved in actin tail formation, cortex destabilization
	RickA	*Rickettsia*	Protrusion	Involved in actin tail formation, WASP mimicry
	Sca2	*Rickettsia*	Protrusion	Involved in actin tail formation, actin nucleator
	Sca4	*Rickettsia*	Protrusion	Involved in protrusion engulfment, interaction with vinculin
	IcsA	*Shigella flexneri*	Protrusion	Involved in actin tail formation, N-WASP activation
	VirA	*Shigella flexneri*	Protrusion	Involved in actin tail formation, microtubule degradation, cysteine protease-like
	MTRAP	*Plasmodium falciparum/berghei* (gametocyte)	Host cell lysis	Involved in PVM rupture, suggested to mediate contact between PVM and parasite cytoskeleton
**Further/unknown**	GEST	*Plasmodium berghei* (gametocyte)	Host cell lysis	Involved in PVM rupture, unknown function
	PAT	*Plasmodium falciparum* (gametocyte)	Host cell lysis	Involved in osmiophilic body discharge
	Transsialidase	*Trypanosoma cruzi* (metacyclic trypomastigote)	Lytic vacuolar escape	Involved in vacuolar escape, unknown function

#### Host cell lysis

**Figure 2:**
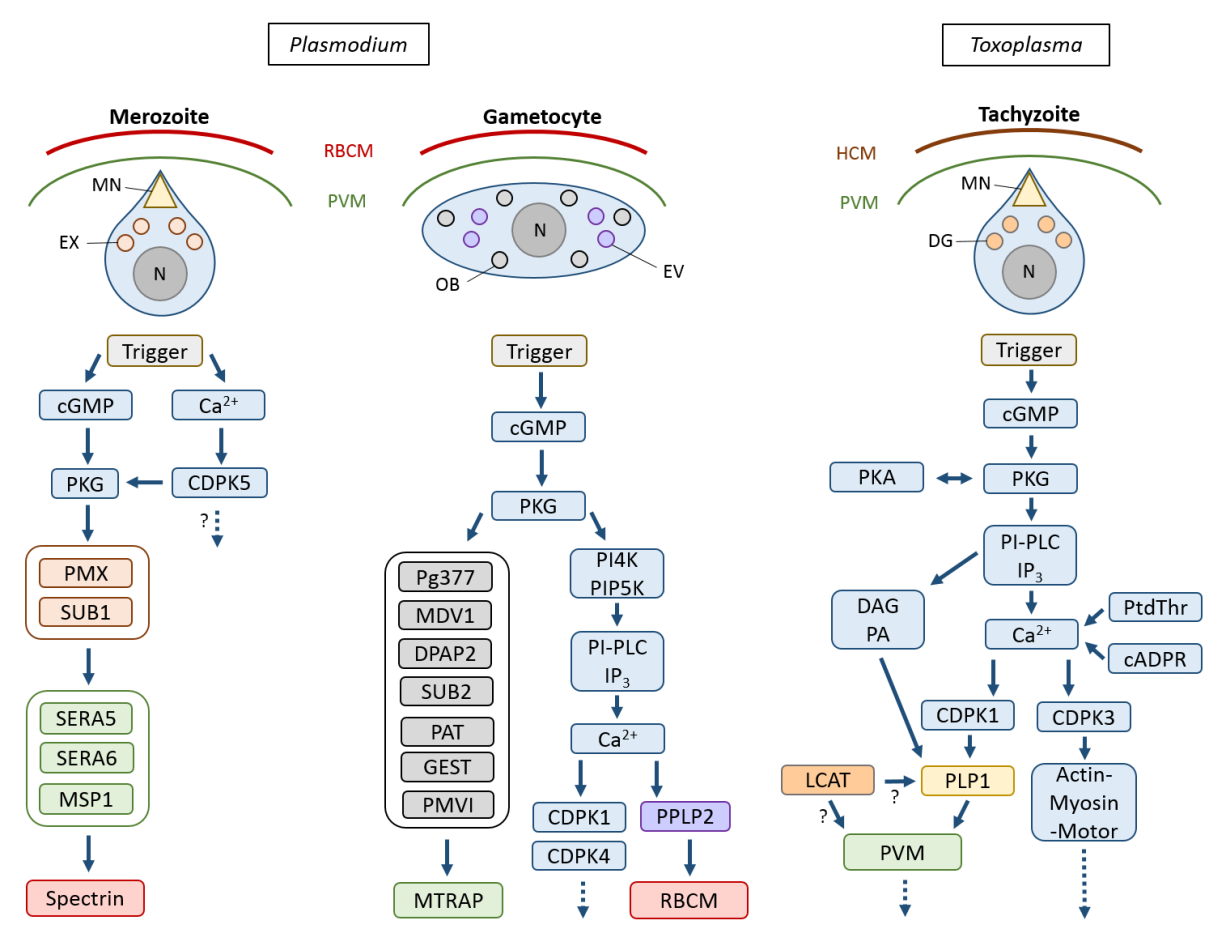
FIGURE 2: Active host cell lysis by Apicomplexan parasites. The key molecules and the sequence of action during host cell egress by merozoites and gametocytes of *Plasmodium* and by tachyzoites of *Toxoplasma gondii*, respectively, are indicated. Question marks indicate steps with ambiguous or unknown interactions. DG, dense granule; EV, egress vesicle; EX, exoneme; HCM, host cell membrane; MN, microneme; N, nucleus; OB, osmiophilic body; PVM, parasitophorous vacuole membrane; RBCM, red blood cell membrane.

Active host cell lysis, which includes the destruction of both host cell plasma membrane and vacuolar membrane, is typical for Apicomplexan parasites and has best been studied for the intraerythrocytic blood and gametocyte stages of malaria parasites, particularly of *P. falciparum*, the causative agent of malaria tropica (Fig. 2). These stages reside within a parasitophorous vacuole (PV) inside red blood cells (RBCs). Following replication in the RBCs, the generated merozoites ultimately have to escape these to disseminate. RBC exit by merozoites follows an inside-out mode, meaning the PV membrane (PVM) ruptures prior to the RBC membrane (RBCM) (reviewed in [6]). The egress cascade begins with the activation of the plasmodial cGMP-dependent protein kinase G (PKG) by a yet unknown signal [33]. Concurrently, the calcium-dependent protein kinase CDPK5 becomes activated due to an increase of intracellular calcium [34, 35]. The concerted activation of both kinases triggers a protease cascade that mediates merozoite egress. Specialized secretory organelles, called exonemes, discharge their content into the PV lumen, including the subtilisin-like protease SUB1 [36]. SUB1 has several targets, like the multiprotein merozoite surface complex called MSP1/6/7 and members of the soluble papain-like PV-resident SERA (sera-repeat antigen) proteins. The aspartic protease PMX, which is also located in the exonemes, mediates the proteolytic maturation of SUB1 [8, 36-39]. In the PV, SERA5 and SERA6 as well as the merozoite surface-resident 200-kDa protein MSP1 become processed by SUB1 [40-42]. Within minutes following the increase of intracellular calcium, the PVM ruptures by a yet unknown mechanism and the RBCM is perforated [43-46]. The processed SERA6, which has β-spectrin cleaving activity, promotes destabilization of the RBC cytoskeleton, while SERA5 exhibits additional regulatory functions [41, 42, 46]. Eventually, the RBCM ruptures at a single breaking point and curls back, a rapid process that disperses the merozoites [47, 48]. Interestingly, merozoite egress from the RBC does not involve actin-myosin motor-driven motility, contrary to sporozoite egress from the oocyst (see below) [49]. RBC exit by merozoites can be inhibited by cysteine and serine protease inhibitors. These include the recently identified PMX-targeting aminohydantoins as well as the hydroxyl-ethyl-amine-based compound 49c, which targets PMIX in addition to PMX [8, 9].

Egress of gametocytes from the RBC occurs at the onset of gametogenesis, once the plasmodia have been taken up by a blood-feeding mosquito, and also follows an insideout mode. In the mosquito midgut, the gametocytes are activated by external stimuli, particularly a drop in temperature and the contact with the mosquito-derived molecule xanthurenic acid (XA) (reviewed in [50-52]). While a plasmodial receptor that binds XA has not been identified, it was shown that gametocyte activation leads to cGMP synthesis and PKG activation [53, 54]. PKG controls the synthesis of phosphatidylinositol-(4,5)-bisphosphate (PIP_2_) via phosphorylation of the phosphoinositol kinases PI4K and PIP5K [55]. A phosphoinositide-specific phospholipase PI-PLC is further activated during the egress cascade, leading to the hydrolysis of PIP_2_ into diacylglycerol (DAG) and inositol-(1,4,5)-trisphosphate (IP_3_) [56]. IP_3_ is suggested to be responsible for the opening of calcium channels in the endoplasmic reticulum, although no plasmodial orthologue of an IP_3_ channel has been identified so far. The calcium ions then regulate two kinases, CDPK1 and CDPK4, which induces the initiation of DNA synthesis in the replicating male gametes and the release of translational repression in female gametes [57, 58]; these events mark the onset of male and female gametogenesis, respectively.

At least two types of vesicles are discharged during gametocyte egress from the RBC. During the first minutes following gametocyte activation, the osmiophilic bodies release their content into the PV lumen. These contain a variety of egress-related proteins, like Pg377, MDV-1/Peg3, the subtilisin protease SUB2, the dipeptidyl aminopeptidase DPAP2, PMVI, the putative pantothenate transporter PAT and the gamete egress and sporozoite traversal protein GEST [59-67]. Consequently, the PVM ruptures at multiple sites. Although the precise mechanism of PVM rupture is undefined, it appears to involve the merozoite thrombospondin-related adhesive protein MTRAP [64, 65, 68]. It was suggested that the membrane-spanning MTRAP links the gametocyte plasma membrane and the PVM and needs to be dismantled prior to PVM rupture, and indeed a rhomboid-protease cleavage site was identified in MTRAP [69]. In a subsequent calcium-dependent step, a second set of vesicles is released into the RBC cytoplasm. These ‘egress vesicles’, which might represent a sub-type of osmiophilic bodies, contain the plasmodial perforin-like protein PPLP2 [63, 64]. Upon discharge, PPLP2 perforates the RBCM, resulting in the release of the RBC cytoplasm [70, 71]. Noteworthy, the male and female gametes that have meanwhile formed are still contained in the remnants of the RBCM for several more minutes, before the RBCM opens via a single pore to release the fertile gametes [71-73]. The delayed cell exit might represent a way to circumvent the host's immune system. Because RBCM breakdown is sensitive to inhibitors of cysteine and serine proteases, it was suggested that the enzymatic cleavage of RBC cytoskeletal proteins precedes membrane rupture [72, 74].

Host cell exit has further been investigated for the Apicomplexan parasite *Toxoplasma gondii*, a feline pathogen that, if taken up by humans, can infect various tissues and causes severe toxoplasmosis, particularly in immuno-compromised individuals (Fig. 2). The lecithin-cholesterol acyltransferase LCAT of *T. gondii* was reported to be released from dense granules, where it associates with the parasite plasma membrane as well as with the PVM prior to egress, suggesting a role in either mediating microneme release or PVM break-down [75, 76]. In addition, the microneme-resident perforin-like protein PLP1 is secreted into the PV, which mediates PVM rupture by forming hexameric pore complexes [77-79]. The micronemes play a central role in host cell exit by *T. gondii* and hence molecules involved in microneme maturation and functionality, e.g. the phosphoglucomutase-related proteins, the micronemal protein MIC2, or the secretory protein ASP3 [80-82], affect parasite egress. Micronemal discharge is stimulated by a variety of factors, which include acidification, serum albumin and the reduction of potassium levels in the host cell cytoplasm [83-85]. Downstream, a PKG becomes activated, in turn regulating PI-PLC activity, which results in increased cytosolic calcium levels [86]. PKG activity is regulated amongst others via cross talk with the protein kinase PKA. Calcium levels appear to be further regulated by the second messenger cyclic ADP ribose (cADPR) via a yet unknown endoplasmic reticulum receptor [87, 88]. It was suggested that cADPR synthesis itself is controlled by the endogenous stress regulator abscisic acid (ABA) [89]; however, neither an ABA receptor nor the signaling pathway induced by ABA has hitherto been identified. In addition, calcium homeostasis is controlled by phosphatidylthreonine (PtdThr) levels, and abrogation of PtdThr synthesis results in the motility-dependent egress of *T. gondii* [90-92]. The increased calcium levels activate the kinases CDPK1 and CDPK3. While CDPK1 is required for microneme secretion, CDPK3 facilitates rapid initiation of motility during parasite egress by phosphorylation of myosin and the suppressor of calcium-dependent egress 1 protein [84, 93-96]. Also involved in micronemal secretion is DAG and its downstream product phosphatidic acid, which is recognized by the microneme-associated acetylated pleckstrinhomology domain-containing protein APH prior to microneme secretion [97].

#### Lytic vacuolar escape

Exit via active destruction has also been described for the escape from the vacuolar compartment by a variety of pathogens, even if the final exit mode follows another route. For example, the plasmodial liver stages use a secreted phospholipase (PbPL) to disrupt the PVM (although the release from infected hepatocytes eventually occurs by budding; see section *Extrusion and budding*) [98]. Noteworthy in this context, prior to settling down in a host hepatocyte contained in a PV, the sporozoites traverse through a variety of tissue cells. Cell traversal initially occurs within a transient (non-parasitophorous) vacuole, which the sporozoite subsequently escapes, a process requiring the the plasmodial perforin PPLP1 [99, 100].

Furthermore, the intracellular Kinetoplastida parasites *Leishmania spp*. and *Trypanosoma cruzi*, which mainly parasitize human phagocytes, are initially contained in a vacuolar compartment of phagosomal and lysosomal origin, respectively, before they escape into the host cell cytoplasm. Their vacuolar escape mechanisms involve parasitederived molecules. For example, *L. amazonensis* expresses leishporin, a protein with membrane-lytic activity [101, 102]. However, a direct proof of active vacuolar lysis by leishporin has not been provided [103]. In addition, the bloodstream trypomastigotes of *T. cruzi* express a transsialidase on their surface, which might mediate vacuolar escape my removing sialic acid from the vacuolar membrane [104, 105]. Further, trypomastigotes secrete a poreforming hemolytic toxin Tc*-*TOX into the vacuole, which is activated by the acidic pH of the lysosomal compartment. It has been proposed that the concerted action of the two proteins mediates rupture of the lysosomal membrane [106, 107]. The detailed mode of action of Tc-TOX, though, is still unknown.

The vacuolar escape of some intracellular bacteria occurs immediately following uptake to evade endosomelysosome fusion and allow replication within the host cell cytosol. This has been shown e.g. for *Shigella, Listeria, Francisella*, and *Rickettsia*. In contrast, bacteria like *Legionella, Mycobacterium*, and *Chlamydia* that remain within the endosomal vacuole manipulate this compartment to inhibit phagosomal-lysosomal fusion. These bacteria have adapted to this modified endosomal compartment and escape the vacuole only shortly before host cell exit (reviewed in [108]). For some of these bacterial pathogens, cytolytic proteins and phospholipases involved in active vacuolar membrane lysis have been reported.

Lytic vacuolar escape has been investigated in detail for the facultative intracellular gram-positive bacterium *Listeria monocytogenes*, which is able to infect a variety of cells including phagocytes. While *L. monocytogenes* enters the host cell within a primary vacuole, the bacterium rapidly escapes the vacuole in order to replicate in the cytosol. Vacuolar escape is essential for intracellular growth, because *L. monocytogenes* replicates inefficiently when contained in vacuoles [109-111]. Cytosolic *L. monocytogenes* uses actin-based motility to spread to the neighboring cells (see section *Actin-mediated protrusion*). Following cell-to-cell transit, *L. monocytogenes* resides in a vacuole enclosed by two membranes, one layer derived from the donor and one from the recipient cell. *L. monocytogenes* also rapidly escapes this secondary vacuole and continuous replicating in the cytosol [112]. To allow endosomal escape, *L. mono-cytogenes* expresses a pore-forming cholesterol-dependent cytolysin, termed listeriolysin O (LLO), which mediates the escape from both, the primary and the secondary vacuoles [113]. Additionally, two phospholipases, PlcA and PlcB, contribute to endosomal membrane disruption [[114, 115]; reviewed in [116]). The two enzymes additionally function in subverting host autophagic defenses by stalling autophagosome formation [117, 118]. The LLO pore-forming activity is enhanced by phagosomal acidification [119-121].

Potential membrane-lytic functions have further been assigned to the type III-secreted translocator proteins IpaB and IpaC of the gram-negative rod *Shigella flexneri*, which causes shigellosis in humans [122-124]. The two proteins form a complex in the vacuolar membrane that binds cholesterol, resulting in membrane degradation. A third protein, IpaD, seems to have regulatory function during this process [125]. Vacuolar escape has also been reported for mycobacteria [126, 127]. Two secreted membrane-lytic proteins, ESAT-6 (a 6-kDa early secretory antigenic protein) and CFP-10 (a 10-kDa culture filtrate protein), were assigned to this process [128-132]. Both proteins are exported into the host cell cytosol through the type VII secretion system ESX-1 (reviewed in [133, 134]); their exact mode of action, however, is still unclear. Vacuolar escape of myco-bacteria rapidly leads to the initiation of PCD (see section *Necroptosis*) [135, 136].

*Chlamydia trachomatis* is an obligate intracellular gram-negative bacterium that infects epithelial cells of the urogenital tract and the conjunctivae. In host cells, the bacteria are contained in vacuolar compartments termed inclusions. *C. trachomatis* can exit the host cell by either extrusion (see section *Extrusion and budding*) or by lysis. Here, *C. trachomatis* first lyses the inclusions by a yet unknown mechanism to escape into the host cell cytoplasm. In preparation for escape, *C. trachomatis* translocates virulence factors into the host cell cytoplasm, among others via a type III secretion system (T3SS). The chlamydial protease-like activity factor CPAF, a serine protease, is initially secreted into the inclusion lumen and eventually crosses the inclusion membrane [137]. Following its release into the cytosol, CPAF processes a wealth of host proteins to promote chlamydial intracellular growth, particularly vimentin and the lamin-associated protein LAP-1 [138]. Chlamydiae lacking CPAF are unable to egress from the host cell [132]. Host cell exit by *C. trachomatis* further requires a variety of plasmid-encoded virulence factors like the transcription regulator Pgp4 [139]. Since cell lysis can be blocked by the cysteine protease inhibitor E64, it has been suggested that cytoskeleton degradation precedes rupture of the host cell membrane [140].

In epithelial cells of the intestine, *S*. Typhimurium resides in a membrane-bound endosome, termed *Salmonella*-containing vacuole (SCV), where it is able to proliferate [141-143]. During the first hours post-invasion, bacteria of a minor proportion of infected cells escape into the cytosol, where they rapidly replicate. The SCV biogenesis involves numerous bacterial effector molecules translocated by the type III secretion systems 1 and 2 (T3SS1 and T3SS2, respectively) (reviewed in [144, 145]). While cytosolic *S*. Typhimurium exhibit T3SS1 expression, T3SS2-positive bacteria remain in the SCV [146]. Noteworthy, poreforming activities were demonstrated for T3SS1 mediated effector molecules and it is thought that the T3SS1 plays a role in damaging the nascent (early) SCV [147-150]. In addition, several T3SS2-translocated effectors like the protein *Salmonella*-induced filaments SifA and SseJ homologous to *L. pneumophila* PlaA (see below) are involved in SCV stabilization and destabilization, respectively, at later time points. SifA counteracts the phagosome rupture by SseJ, which shows PLA and cholesterol acyltransferase activities [151-153]. SifA binds to SKIP (SifA and kinesin-interacting protein), which in turn interacts with kinesin-1 and the secreted effector protein PipB2. This interaction might regulate SCV stability [154-156].

*Legionella pneumophila*, a facultative intracellular bacterium, infects amoeba under environmental conditions. Once entering the human lung, it can invade macrophages, thereby causing severe lung infections. In both, macrophages and amoebae, *L. pneumophila* replicates in a specialized phagosome avoiding fusion with the host endocytic pathway. A type IVB secretion system (Dot/Icm) responsible for the translocation of a multitude of effector proteins into the host cell as well as the type II secretion system Lsp are required for intracellular replication (reviewed in [157]). *L. pneumophila* exits from the phagosome either by nonlytic exocytosis (see section *Exocytosis, expulsion and ejection*) or via a pore-forming activity [158-161]. Pore formation might be triggered by phospholipases found in *L. pneumophila* (reviewed in [162]). Specifically, the type II secreted lysophospholipase PlaA, showing homology to *S*. Typhimurium SseJ (see above), destabilizes the phagosomal membrane in the absence of the type IVB-secreted protective factor SdhA [163, 164]. The detailed mode of phagosome lysis remains to be investigated.

A special form of lysis-mediated microbial exit is the one from cysts. This has been investigated for the egress of *P. berghei* from oocysts, parasite-derived cyst-like structures, which form after sexual reproduction and are found in the mosquito midgut attached to the epithelium. Oocyst exit required two proteases, *P. berghei* SERA5 (an orthologue of *P. falciparum* SERA8) and PMVIII [165, 166]. Other plasmodial proteins that mediate sporozoite maturation, e.g. the thrombospondin-related protein TRP1 or the LCCL-domain containing proteins, were further assigned to oocyst egress by malaria parasites [167-170]. Noteworthy, the motility of *Plasmodium* sporozoites, driven by an actinmyosin motor, is necessary for oocyst egress [170].

### Induced membrane-dependent exit

Some microbes are able to leave the intact host cell. Such exit strategies include actin-driven membrane protrusions enabling the spread of single bacteria between cells, extrusions and budding of microbes packed in a membranous compartment as well as ejection, expulsion and exocytosis of free microbes (Fig. 1). The detailed mechanisms of induced membrane-dependent exit, however, are to date not well defined and only few key molecules of this exit pathway have been identified (Table 1).

#### Actin-mediated protrusion

Polar recruitment and polymerization of actin results in a directed locomotion of cytosolic bacteria. Actin polymerization is facilitated by the expression of a microbial surface protein that binds or mimics the host cell actin-related protein (Arp)2/3 complex [171]. Reaching the cell border, the advancing bacterium is able to protrude the host cell plasma membrane by physical force. Since bacteria are unable to penetrate this membrane, they project from the infected cell within the tip of a filopodium-like membrane extension. This cell membrane protrusion is subsequently engulfed by the adjacent cell [172]. Notably, this cell-to-cell spread occurs without contact to the extracellular environment and thus protects the pathogen from exposure to extracellular immune surveillance mechanisms and antimicrobial effector molecules. The most intensively studied microorganisms performing actin-mediated protrusion are *Listeria monocytogenes* and *Shigella flexneri*, but a similar ability has been demonstrated for the spotted fever group of rickettsiae, *Burkholderia spp., Ehrlichia spp*. and *Myco-bacterium marinum*.

Upon release from the endosomal membrane, *L. monocytogenes* starts polar expression of the surface molecule actin assembly-inducing protein (ActA) [173]. The N-terminus of ActA represents a mimic of the naturally occurring actin nucleation-promoting factor Wiscott-Aldrich syndrome protein (WASP) and binds preformed Arp2/3 complexes [174]. The central domain of ActA additionally binds profilin and vasodilator-stimulated phosphoprotein (VASP), promoting the rapid formation of branched actin polymers. Furthermore, parallel actin polymerization and actin tail assembly is facilitated by the activation of Rho GTPase and the actin polymerization factor formin [112, 175]. The actin comet tail thereby propels the bacterium through the host cell cytosol. Once approaching the vicinity of the plasma membrane the secreted *Listeria* protein internalin C (InlC) binds to the host adaptor protein Tuba to inhibit the actin polymerization promoting neural WASP (N-WASP) and to the COPII complex component SEC31 [176]. This weakens the cortical tension at the plasma membrane allowing *Listeria* to protrude the plasma membrane in a filopodia-like fashion [175]. The membrane cytoskeleton linker ezrin accumulates at the protrusion site and stabilizes the growing membrane extension [177]. While it is still unclear, how exactly the bacterium-containing membrane extension is engulfed by the adjacent cell, the process requires active participation of both, the donor and recipient cell. For example, the host casein kinase CSNK1A1 was shown to promote the conversion of protrusions to endosomes in the donor cell [178, 179]. After transfer, *Listeria* resides in an endosomal compartment composed of a membrane layer of both the donor and recipient cell. This double-layered endosomal membrane is lysed by PlcB, PlcA, and LLO releasing the bacterium in the recipient cell cytosol (see section *Lytic vacuolar escape*) [115, 180-183]. Importantly, the ability to perform cell-to-cell spread represents a critical component of *L. monocytogenes* virulence and does not lead to host cell death [181, 184, 185].

Actin-based motility also critically contributes to *Shigella flexneri* virulence [186]. Here, the *Shigella* IcsA protein activates N-WASP to recruit Arp2/3 and initiate actin tail formation [187-189]. Simultaneously, *Shigella* stimulates RhoA GTPases and the mammalian diaphanous-related formins mDia1 and mDia2 to promote actin polymerization in parallel arrays at the protrusion site [190]. The virulence factor VirA of *S. flexneri* degrades the cytoplasmic microtubule network via its cysteine protease-like activity and might thereby promote cytosolic locomotion [191, 192]. Similar to *Listeria*, engulfment of the *Shigella*-containing protrusions by the adjacent cell does not depend on additional bacterial factors. Instead, it requires active participation of the host cell proteins. The requirement for tricellulin, epsin-1, clathrin and dynamin-2 suggest the involvement of a noncanonical clathrin-dependent endocytic pathway in the recipient cell [193, 194].

Other bacteria that employ actin-based motility to allow cell-to-cell transfer include the spotted fever group rickettsiae such as *Rickettsia rickettsii, R. conorii* or *R. parkeri* [195]. The molecular basis, however, is less well examined. Early motility has been attributed to polar expression of RickA, which recruits the Arp2/3 complex and induces actin polymerization in an N-WASP-like manner. Later, motility occurs in an Arp2/3 independent fashion via secretion of the autotransporter surface cell antigen (Sca) 2, a formin-like actin nucleator that generates long unbranched actin tails [196, 197]. Subsequent secretion of Sca4 promotes protrusion engulfment through interaction with the cell-adhesion protein vinculin. This relieves intercellular tension and the engulfment of bacteria-containing protrusions [198].

*Ehrlichia chaffeensis* recruits N-WASP to generate actin polymerization and form bacteria-driven filopodia and allow cell-to-cell spread [199, 200]. The pathogenesis of *Burkholderia spp*. relies on actin-based motility [201]. Whereas the secreted trimeric autotransporter BimA (*Burkholderia* intracellular motility A) of the animal pathogen *B. thailandensis* activates the host Arp2/3 complex, the orthologue BimA of the human-pathogenic *B. pseudomallei* and *B. mallei* mimic host Ena/VASP actin polymerases [202]. In contrast to the above-discussed pathogens, cell-to-cell spread is facilitated by fusion of infected and adjacent cells induced by the type VI secretion system (T6SS)-1 [203]. *Mycobacterium marinum*, a natural fish pathogen and occasional human pathogen, requires either WASP or N-WASP to perform actin-based motility and both the Arp2/3 complex and the vasodilator-stimulated phosphoprotein were identified as constituents of the actin tail [126, 204]. The molecular mechanism of subsequent cell-to-cell spread may differ from the other pathogens.

#### Extrusion and budding

Host cell exit via extrusion or budding involves the release of membrane-encircled microorganisms (Fig. 1). Here, the membrane coat protects the microbe against humoral factors of the host immune system. To date, only few molecular players have been identified that modulate this type of host cell exit (Table 1).

Extrusions have been reported for *Chlamydia trachomatis*. Chlamydial extrusions depend on actin polymerization mediated by N-WASP and Rho GTPases, while myosin and septins are involved in regulation and stabilization of the actin filaments [140, 205]. A bacterial protein appears to recruit the myosin-activating machinery to the inclusion to favor extrusion of pathogens over a cell-lytic pathway [206].

Viral-like budding was shown for the scrub typhus-causing bacterium *Orientia tsutsugamushi* that replicates in the cytosol of a variety of host cells, including phagocytes. Inside its host cell, the bacterium is ‘encapsulated’ by the plasma membrane of the cell. Host cell exit appears to depend on lipid rafts and a bacterial protein that was found to co-localize with caveolins at the site of cell exit, suggesting a role in egress ([207], reviewed in [208]).

Host cell exit by budding was further demonstrated for the intrahepatic merozoites of *Plasmodium*. Following phospholipase-mediated PV breakdown (see section *Lytic vacuolar escape*), these lie in the hepatocyte cytoplasm and are subsequently released into the blood stream. The merozoites leave the hepatocyte in host cell plasma membrane-derived vesicles termed merosomes that can contain a few up to several hundreds of merozoites [209]. The parasites induce the separation of the actin cortex from the hepatocyte plasma membrane prior to merozoite formation [210]. The molecular mechanisms that allow the vesicle to bud from the hepatocyte and penetrate the endothelial cell layer to reach the blood stream are, however, unknown. Interestingly, although PCD is induced by the parasite in the infected hepatocyte, the budding merosomes do not expose phosphatidylserine on their surface as would be expected from apoptotic cells [211]. This probably helps the merosomes to remain undetected by macrophages as they leave the liver and travel to the lung tissue [212]. Contrary to the induced membrane-dependent exit by other intracellular pathogens, in this case, the host hepatocyte dies.

#### Exocytosis, expulsion and ejection

Exocytosis involves the transport of molecules from intracellular endosomal vesicles to the plasma membrane. The membrane of transport vesicles fuses with the plasma membrane to allow cargo release and thereby aids the passage of mostly large and polar substances into or through the plasma membrane (reviewed in [213, 214]). A variety of pathogenic microbes exit host cells by exocytosis and exocytosis-like expulsion (also called vomocytosis) as well as by mechanistically distinct ejection. All these procedures have in common that they release free pathogens without harming the host cell. The processes therefore avoid the release of proinflammatory cellular constituents (reviewed in [4, 5, 215]).

Exocytosis-like egress from amoebae has been described for *Legionella pneumophila*. Within the host cell, *L. pneumophila* is contained in a specialized phagosome and via a type IVB secretion system secretes effector proteins into the host cell cytoplasm to allow replication (see section *Lytic vacuolar escape*) (reviewed in [157]). Such effectors frequently harbor signatures of eukaryotic proteins and some of these exert homology to the SNARE (soluble N-ethylmaleimide-sensitive factor attachment protein receptor) proteins known to mediate the fusion of vesicles to biological membranes [216-219]. These analogous bacterial effectors may drive or even abrogate vesicle fusion events. Two type IVB-secreted *L. pneumophila* proteins, LepA and LepB, with regions weakly similar to mammalian early endosome antigen 1 (EEA1), required for endosome docking of SNARE proteins, were identified. They play a critical role in the bacterial release from the amoebae *Acanthamoeba castellanii* or *Dictyostelium discoideum* [216], indicating that *L. pneumophila* is released from amoebae by an exocytotic pathway. However, earlier reports described vesicle-associated *Legionella* expelled from *A. castellanii* [220]. This raises the question whether the bacteria are indeed released by the above-described process in their free form or rather in a vesicle-enclosed form.

Non-lytic egress of the fungus *Cryptococcus neoformans* is debated as an important factor determining systemic dissemination of the pathogen [221, 222]. The yeast exerts an exocytosis-like expulsion, called vomocytosis, in macrophages. Non-lytic expulsion of *Cryptococcus* occurs through fusion of the phagosome and plasma membrane. Mutants defective for the secreted phospholipase PLB1 or the PLB1-exporting secretion system Sec14 exhibit reduced quantities of vomocytosis. The actin cytoskeleton of the host cell is not essential for this process [223-226]. However, macrophage phagosomes containing intracellular cryptococci undergo repeated cycles of actin polymerization, called actin ‘flashes’ dependent on the classical WASP-Arp2/3 complex. Prior to expulsion, the majority of phagosomes is permeabilized, which is immediately followed by an actin flash likely devoted to temporarily inhibit expulsion [225]. Vomocytosis has also been observed upon *in vivo* infections of mice and zebrafish [227, 228]. The latter study reported that inhibition of the mitogen-activated protein kinase ERK5 increased vomocytosis and decreased pathogen dissemination, indicating that vomocytosis enhancement might represent a therapeutic target.

A particular mode of cell exit was discovered in *Mycobacterium tuberculosis* and *M. marinum*. Both are non-lytically ejected from *D. discoideum* through an F-actin-based structure, the ejectosome [229]. The process involves the so-called region of difference RD1 locus, where components of the mycobacterial type VII secretion system ESX-1 are encoded [127, 130, 135, 229, 230]. Both the ESX-1 secretion system and its secreted effector ESAT-6/EsxA are required for mycobacterial ejection from *Dictyostelium* and bacterial translocation into the cytosol of mammalian cells. A recent report describes an unexpected role of the autophagic machinery in non-lytic release of *Mycobacteria* and cell-to-cell transmission in *Dictyostelium* [231]. Further, the extraordinary importance of maintaining membrane integrity during the process of ejection is highlighted. Specifically, the study indicates that bacteria shortly prior to ejection are escorted by an autophagocytic vacuole, which is recruited in an ESX-1 independent manner. When autophagy is impaired, cell-to-cell transmission is inhibited. In this case, the host plasma membrane becomes compromised and the host cell subsequently dies [231]. These findings illustrate that non-lytic egress by ejection requires host cell-derived membrane protection pathways.

## HOST CELL EXIT BY INTRACELLULAR PATHOGENS: THE ACHILLES’ HEEL?

The combined data highlighted in this review suggest that host cell exit by intracellular pathogens represents a fundamental and active step in infection, which, shaped by evolutionary pressure, is crucial for microbial spread and might represent the Achilles' heel of microbial pathogenesis. A limited set of host cell exit pathways appears to be shared by a high variety of phylogenetically different microbes with the involvement of similar types of pathogen-derived proteins, like proteases, pore-forming proteins and phospholipases or actin-binders. This strongly suggests convergent evolution of the exit machineries. The fact that exit strategies employ manipulated secretion or delivery routes as well as the complex cytoskeletal restructuring further point to the intimate interaction between pathogens and their host cells.

While much experimental work lies ahead of us to decipher the molecular mechanisms of host cell exit by intracellular pathogens, evidence emerges that key molecules of host cell exit are promising targets for novel types of interventional strategies. The fact that only few classes of pathogen-derived proteins appear to be involved in the exit processes and that in general their accessibility for inhibitors is known makes host cell exit as a point of attack even more attractive. Indeed, as described earlier, first protease inhibitors have been identified that are able to block the egress of malaria parasites from RBCs [8, 9]. While targeting microbial exit might not protect from primary infection, the cell-entrapment of microbes ensures immediate control of microbial tissue spread and disease progression. Importantly, it should still allow stimulation of a protective adaptive immune response thus providing therapeutic and prophylactic advantages. Such approaches have been exploited in the recent past for liver stage-targeting antimalarial vaccines, using attenuated parasites (reviewed in e.g. [232-234]) and might represent a pioneering strategy to combat life-threatening human infectious diseases. Concluding, present pieces of evidence point to exit strategies of intracellular pathogens as an emerging field of infection biology essential to fully understand and successfully counteract microbial pathogenesis.

## Abbreviations:

ABA: – abscisic acid,
ActA: – actin assembly-inducing protein,
Arp2/3: – actin-related protein 2/3,
CPAF: – chlamydial protease-like activity factor,
N-WASP: – neuronal WASP,
PCD: – programmed cell death.
PI: – phosphatidylinositol,
PK: – protein kinase,
PV: – parasitophorous vacuole,
PVM: – PV membrane,
RBC: – red blood cell,
RBCM: – RBC membrane,
WASP: – Wiscott-Aldrich syndrome protein.
